# Generation of Digital Brain Phantom for Machine Learning Application of Dopamine Transporter Radionuclide Imaging

**DOI:** 10.3390/diagnostics12081945

**Published:** 2022-08-12

**Authors:** Wenyi Shao, Kevin H. Leung, Jingyan Xu, Jennifer M. Coughlin, Martin G. Pomper, Yong Du

**Affiliations:** 1 The Russell H. Morgan Department of Radiology and Radiational Science, Johns Hopkins University School of Medicine, Baltimore, MD 21287, USA; 2 Department of Biomedical Engineering, Johns Hopkins University School of Medicine, Baltimore, MD 21287, USA; 3 Department of Psychiatry and Behavioral Sciences, Johns Hopkins University School of Medicine, Baltimore, MD 21287, USA

**Keywords:** deep learning, generative adversarial network (GAN), phantom, SPECT

## Abstract

While machine learning (ML) methods may significantly improve image quality for SPECT imaging for the diagnosis and monitoring of Parkinson’s disease (PD), they require a large amount of data for training. It is often difficult to collect a large population of patient data to support the ML research, and the ground truth of lesion is also unknown. This paper leverages a generative adversarial network (GAN) to generate digital brain phantoms for training ML-based PD SPECT algorithms. A total of 594 PET 3D brain models from 155 patients (113 male and 42 female) were reviewed and 1597 2D slices containing the full or a portion of the striatum were selected. Corresponding attenuation maps were also generated based on these images. The data were then used to develop a GAN for generating 2D brain phantoms, where each phantom consisted of a radioactivity image and the corresponding attenuation map. Statistical methods including histogram, Fréchet distance, and structural similarity were used to evaluate the generator based on 10,000 generated phantoms. When the generated phantoms and training dataset were both passed to the discriminator, similar normal distributions were obtained, which indicated the discriminator was unable to distinguish the generated phantoms from the training datasets. The generated digital phantoms can be used for 2D SPECT simulation and serve as the ground truth to develop ML-based reconstruction algorithms. The cumulated experience from this work also laid the foundation for building a 3D GAN for the same application.

## 1. Introduction

Nuclear medicine imaging, including both single-photon emission computed tomography (SPECT) and positron emission tomography (PET), is an important molecular imaging tool for studying neurological disorders, such as degeneration of nigrostriatal dopaminergic neurons in patients with Parkinson’s disease (PD) [1,2,3]. Nuclear medicine is also used for the diagnosis and monitoring of many other diseases such as cardiac vascular diseases, cancer, etc. [4,5,6]. However, the spatial resolution of PET images is limited to 3–6 mm due to the imaging physics [7]. SPECT imaging is also known to suffer from even poorer sensitivity and resolution (1–2 cm full width half maximum (FWHM)) due to the use of collimators [8]. The resulting data is thus blurred and contains high noise, which makes image reconstruction and subsequent analysis challenging.

Currently, artificial intelligence (AI) and machine learning (ML)-based methods have been widely applied to medical imaging. Typical examples include magnetic resonance imaging (MRI) image reconstruction [9] and compressed sensing [10,11], sparse-view computer tomography (CT) reconstruction [12,13,14], PET image reconstruction [15] and attenuation correction [16,17,18], SPECT attenuation map generation [19] and image reconstruction [20,21,22], etc. ML-based solutions may help overcome some of the physics or hardware limitations in the sense that the result provided by ML is not 100% reliant on the measurement data alone, but is also partially based on the experience obtained from massive training with known ground truth. However, clinical data is usually limited in quantity (with unknown truth). Therefore, most of the existing ML algorithms for nuclear medicine imaging are trained by merely tens to hundreds of patient datasets and validated by even fewer datasets. As a result, these ML models are likely to be overfitted to the limited datasets instead of providing a general solution to the problem under consideration.

A typical example was by Hwang in 2018 [16], in which PET images reconstructed by ordered subset expectation maximization (OSEM) algorithm from only 40 patients were used as the ground truth for training an AI algorithm. Moreover, when reconstructed images are used as the target to develop an AI reconstruction algorithm, the acquired model can merely produce images with quality matching the traditional algorithms (like OSEM), which is already ubiquitously available in clinical systems. In order to make full use of AI’s capability to reconstruct better image, a large dataset with known truth is needed. This can be obtained through simulations that uses accurate physics modelling such as Monte Carlo simulation [23,24,25]. However, current existing digital phantoms such like Zubal phantom [26] and XCAT phantoms [27] are usually generated from a single person’s anatomy and lack anatomical variations present on a population level. Thus, there is an urgent need to develop a large population of digital phantoms that model anatomy variations seen in clinic.

To resolve this, some AI imaging scientists have settled on using natural images (e.g., Imageset, which contains tens of thousands of natural images such as cats, deserts, vehicles, etc. as phantoms) to generate clinical data by simulation, and by which an AI medical image reconstruction system was then developed [9]. This is a fundamentally flawed solution because natural images do not contain information about human anatomy, diseases, and radioactivity distribution (in addition, SPECT was not investigated in [9]). Recently, generative adversarial network (GAN) has been demonstrated for its ability in data augmentation in order to achieve better performance in AI-based CT imaging [28]. Motivated by this, the present work develops a method that uses GAN to produce many digital brain phantoms that mimic the imaging of dopamine transporter and the attenuation map with high resolution. The generated phantoms will contain activity distributions and attenuation maps to reflect anatomical and uptake variations that are commonly seen clinically in PD. Although GAN has been adopted to develop medical anatomical phantoms for MRI [29], CT [28] and in microwave medical imaging [30,31] research, to our best knowledge, the current paper is the first time that biomarker-distribution phantoms and attenuation maps have been developed by the GAN technique. With the generative network, unlimited number of phantoms with more variations can be produced, which will then be used to develop more robust AI-based PD SPECT imaging algorithms than former AI models [20,21,22,32]. Although the present paper is limited to 2D, experience cumulated in this work will lower the difficulty of designing more complex GANs to produce 3D brain phantoms in the next step.

## 2. Data and Methods

### 2.1. Training Datasets Preparation

The training data for developing the GAN came from 155 3D PET brain models (113 male and 42 female) downloaded from Parkinson’s progression markers initiative (PPMI) website [33]. No healthy volunteers’ datasets were available as we downloaded the data, thus all datasets adopted in this article are assumed to be from suspected PD patients (different disease stages are possible). A feature of GAN, that it can extend a large population synthetic data from a small source-data pool, determines that it is not necessary to have a large training-data pool, which is critical in the usual development of deep-learning models. Therefore, we considered that the amount of patient data we collected was sufficient for developing GANs (but not for developing usual deep-learning models) in this work.

**Figure 1:**
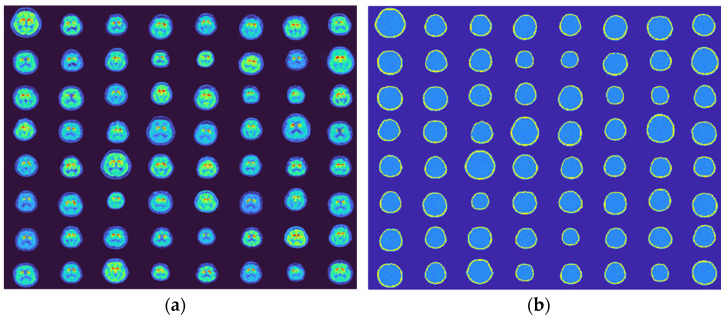
Digital phantoms to be used to train the GAN. Images are slices containing striatum or a portion of the striatum selected from 3D models. (**a**) PET (activity) images and (**b**) generated corresponding attenuation maps.

After careful review, 1597 2D trans-axial slices that contained at least a portion of the striatum were selected. The reason of using PET images instead of SPECT images is due to higher resolution in PET images, such that a future AI-based SPECT imaging system can be expected to achieve the same (or a close) resolution as PET. Despite SPECT using different imaging biomarkers (e.g., [^123^I] ioflupane) than PET ([^18^F] FDG), the PET images can be imported to a SPECT Monte Carlo simulation to acquire SPECT signals. Hence, an image reconstruction AI model will still be able to learn the mapping between brain radioactivity images and SPECT signals. The thickness of the 2D layer was slightly different among all slices because the 3D models were collected by different machines and research institutions. However, they will be assumed to be the same in the present work because this article only focuses on 2D. The attenuation maps were generated by defining the soft tissue with the same attenuation coefficient as water (plus up to ±10% to represent the inhomogeneous) in the attenuation maps, and adding a layer of skull according to the edge of the brain. A selection of phantoms including the activity images and their corresponding attenuation maps are presented in Figure 1. Each sub-image in Figure 1 has 128 by 128 pixels.

### 2.2. Neural Network Details and Training

**Figure 2:**
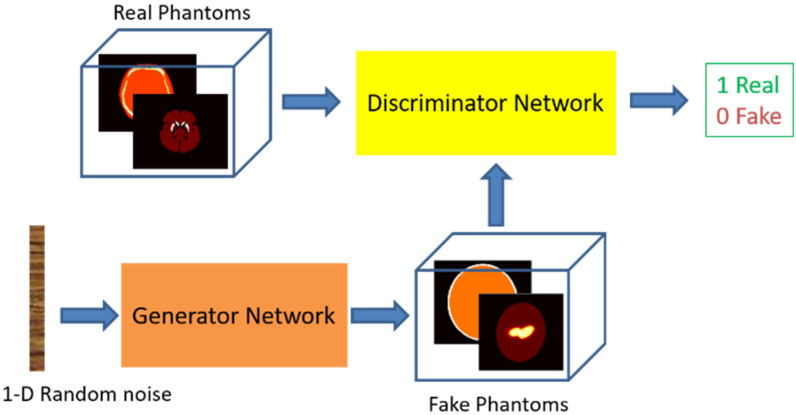
The training scheme of GAN for generating numerical brain phantoms for the PET/SPECT PD study. Each phantom is composed of two images: one representing the radiopharmaceutical distribution in the brain and the other representing a corresponding attenuation map. The fake phantoms denote the generated image.

GAN is a type of deep neural network that can generate data with similar characteristics as the given training data but with more variations. A GAN consists of two neural networks that are trained together: a generative network (generator), which generates new data given a random signal as the input; and a discriminative neural network (discriminator), which evaluates the data for authenticity. That is, the discriminator attempts to classify the observation belonging to the training dataset (real) or the generated dataset (fake). By alternately training the generator and the discriminator in steps, the discriminator gradually learns strong feature representations that are characteristic of the training data, while the generator can generate convincing data that are recognized by the discriminator as real. The scheme for training such a GAN system for phantom generation purposes is illustrated in Figure 2. The goal is that, when a generated phantom is delivered to the discriminator, the output of the discriminator is or close to 0.5, meaning that it is difficult to identify whether the input belongs to the training dataset or a generated dataset. Two networks are trained simultaneously to maximize the performance of both.

**Figure 3:**
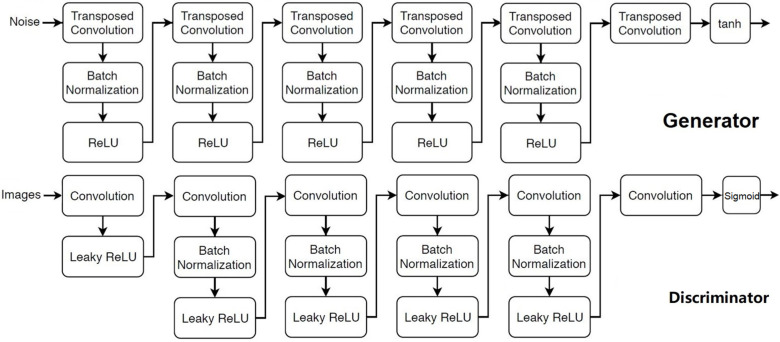
The network architecture of the generator (up) and discriminator (low). The number of filters from the first transposed convolution layer to the last in the generator is 1024, 512,256, 128, 64, 2, respectively. Filter size is 4 by 4 in all layers. The number of filters from the first convolution layer to the last in the discriminator is 64, 128, 256, 512, 1024, and 1. Filter size was 5 by 5 in all layers except in the last convolution layer, which was 4 by 4.

The generator is comprised of six 2D transposed convolutional layers with each followed by batch normalization and a rectified linear activation function (ReLU), except for the last transposed convolutional layer that is followed by a hyperbolic tangent function, to convert the input consisting of 100 random numbers to an image of a pair of 128 by 128 voxels. The discriminator consists of six 2D convolutional layers, each followed by a batch normalization layer and a leaky ReLU function:

(1) $$f\left(x\right)=\left\{\begin{array}{c} x,\;\;\;\;\;\;\;\;\;\;\;\;\;\;\;\;\;\;\;x\geq 0\\ 0.2\times x,\;\;\;\;\;\;\;x<0 \end{array}\right.$$

except in the first convolutional layer where no batch normalization was used, and in the last convolutional layer which is only followed by a sigmoid function. The stride in the convolution layer (discriminative network) and the transposed convolution layer (generative network) is 2. The network structure of the generator and discriminator is presented in Figure 3.

To allow the networks to converge to a better solution, the training dataset was normalized before being given to the discriminator. In the activity image and attenuation map, pixels that belonged to the brain region were linearly converted to the range from 0 to 1 (positive numbers). Pixels that were out of the brain region were all set to negative (−1). Thus, the total output range of the generative network is −1 to 1, which is consistent with the output range of a hyperbolic tangent function (the final activation function of the generative network). It is estimated that a well-trained generator would not necessarily be able to produce images with background pixels all as −1, but they will most likely be negative numbers. So, it will be easy for us to recognize the profile of the brain, i.e., yielding a sharp edge of the brain.

All hyper-parameters such as the mini-batch size, filter size, etc., have been optimized via multiple trials. Since the training of the generator relied on what it had learned from the discriminator, if the discriminator learns too quickly, the generator may fail to follow up. Hence, the learning rate of the generator was set to 0.0001 and the learning rate of the discriminator had to be smaller, which was set to 0.00006 in our case. The Adam optimization algorithm [34] was applied to both networks, for which algorithm the gradient decay factor was set to 0.5 and the squared gradient decay factor was 0.999. The objective of the generator was to generate data that the discriminator classified as “real”. To maximize the probability that images from the generator were classified as real by the discriminator, we minimized the negative log-likelihood function. Thus, the loss functions of the generative network were given by

(2) $$loss_{g}=-\frac{1}{M}\sum \log P_{g}$$

where $\;P_{g}\;$ is the discriminator’s estimate of the probability when generated phantoms were passed to the discriminator, and *M* is number of observations, usually the mini-batch size. The objective of the discriminator is to not be “fooled” by the generator. To maximize the probability that the discriminator successfully discriminates between the real and generated phantoms, we minimized the sum of the corresponding negative log-likelihood functions. Thus, the loss function of the discriminator was given by

(3) $$loss_{d}=-\frac{1}{M}\sum \log P_{r}-\frac{1}{M}\sum \log\left(1-P_{r}\right)$$

where $P_{r}$ is the discriminator’s estimate of the probability when real phantoms were given to the discriminator.

### 2.3. Optimization of Training

A method of adding noise to the training data may help converge the networks (the networks were actually found to fail to converge if not doing so, in this case). However, instead of adding Gaussian white noise to the training dataset directly [35], we introduced the noise by randomly flipping the labels (of real images) for the discriminator. For example, when the flip factor was set to 20%, then 10% of the total number of labels were flipped (meaning 10% real were set to fake, and 10% generated image were set to real) during the training. This is done by using

(4) $$P_{r}=1-P_{r}$$

for those randomly selected real data. The flip factor worked as a parameter that can be easily tuned in the program. Note that this does not impair the generator as all the generated images are still labelled correctly. The flipping rate was gradually decreased during the training and diminished in the late stage to exclude the label noise.

The training was executed on a Nvidia P6000 GPU and was manually terminated after running 4800 iterations, which took 2.5 h according to the evolution of the loss in the discriminator and generator in conjunction with statistical evaluation to be discussed in the next section.

## 3. Results

### 3.1. Generated Phantoms

Since there are no fully connected layers in the generator, the generator has only 12,783,554 learning parameters, leading to a very compact storage size (approximately 50 megabytes, single precision). The generative network generates 2D phantom with 128 × 128 voxels with normalized data, so the data will need to be converted back to the original space, using the following formula for both the activity images and attenuation maps:

(5) $$I^{\prime }\left(\vec{r}\right)=\left\{\begin{array}{l} 0\;\;\;\;\;\;\;\;\;\;\;\;\;\;\;\;\;\;\;\;\;\;\;\;\;\;\;\;\;\;\;I\left(\vec{r}\right)<0\\ LT\left(I\left(\vec{r}\right)\right)\;\;\;\;\;\;\;\;\;\;I\left(\vec{r}\right)\geq 0\;\; \end{array}\right.$$

where $I(\vec{r})$ represents the pixel value in the normalized image (output of the generator), $I^{\prime }(\vec{r})$ stands for the new pixel value after the conversion, and LT stands for a linear transfer back to the original data space.

**Figure 4:**
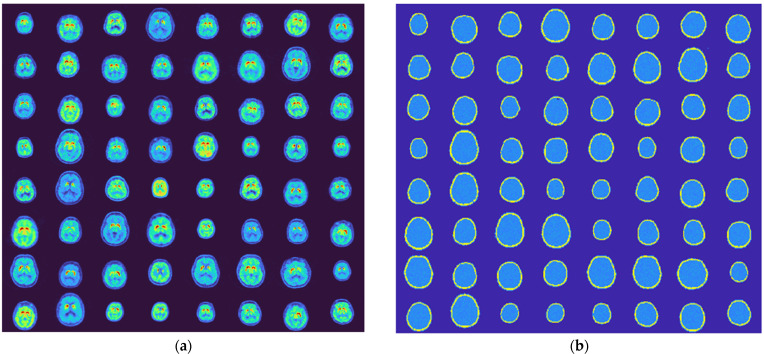
Generated phantoms by the developed generator. (**a**) Generated activity maps and (**b**) generated corresponding attenuation maps. Each sub-image has 128 by 128 pixels.

The generator was employed to produce 10,000 brain phantoms, which took approximately 1 h on a Dell Precision workstation with a 3.0 GHz Xeon CPU (using single thread). A computer method (MATLAB SSIM function) was performed to verify that each phantom was unique in the generated database, which somehow demonstrated the diversity of the generated data. Figure 4a shows some generated activity images and Figure 4b shows the corresponding attenuation maps, when (5) has been executed. Each sub-image has 128 × 128 pixels. Each image pair represents a 2-D phantom, i.e., a trans-axial slice that contains at least a portion of the striatum. As seen, the generated images are very comparable to the training data presented in Figure 1.

### 3.2. Evaluation of the Phantoms

**Figure 5:**
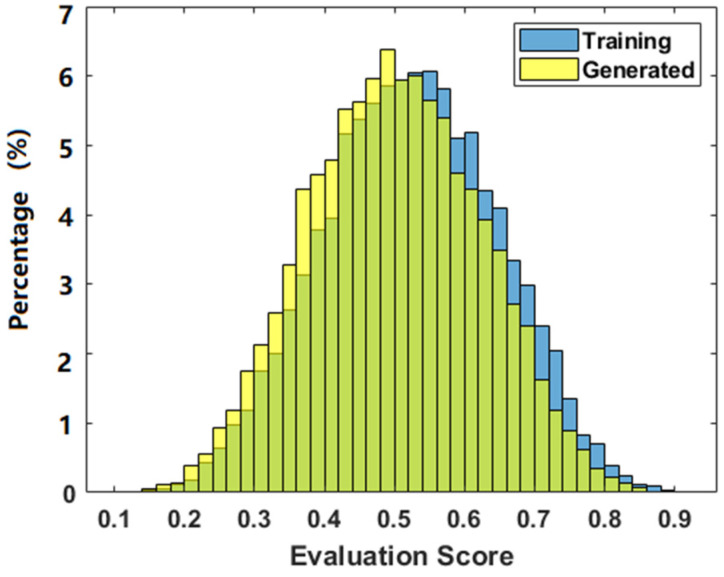
The distribution of the phantoms. Yellow bars represent the frequency of generated phantoms, and blue bars represent the frequency of the training phantoms.

The generated phantoms are difficult to be evaluated by common methods such as mean square errors. Manual inspection can be a qualitative assessment approach, but is subjective, including biases of the reviewers, and is also limited to the number of images that can be reviewed within a reasonable time. Quantitative assessment of GAN models remains an open problem today. Nevertheless, the discriminator that was trained in tandem with the generator is actually a good tool for quantitatively evaluating the generated phantoms, while the discriminator is often neglected when training is complete, since the generator is what is desired. In this work, the generator was used to produce 10,000 phantoms, which were then passed to the discriminator for a statistical evaluation. Note that what were passed to the discriminator are the images before performing the transfer by Equation (5). The discriminator output denotes the score of the phantom, i.e., the probability of the generated phantom belonging to the training dataset. The histogram of the phantom scores is presented in Figure 5. As a result, the scores of the generated phantoms were in a normal distribution with a peak at the range of 0.48 to 0.50, and 648 phantoms fell in this range (6.48% of the total 10,000 generated phantoms). The average score of all 10,000 generated phantoms was 0.4983, which is very close to 0.5. The minimum was 0.1186 and the maximum was 0.8676.

To compare the generated phantoms with the training datasets, we forwarded all 1597 training datasets to the discriminator. The average score of all training datasets was found to be 0.5237, the minimum score was 0.1476 and the maximum was 0.9059. The histogram of the training dataset is shown in blue bars in Figure 5, which presents a normal distribution with a peak appearing at 0.54 to 0.56, where 303 phantoms fell in this range (6.12% of the total 4992 training phantoms).

In addition to visually comparing of the distributions, another quantitative tool often used to compare the similarity of two groups of the probability distribution, Fréchet distance (FD) [36], was employed to compare the two distributions. The FD is defined by

(6) $$d^{2}=\left|µ(r)-µ(g)\right|{\;}^{2}+Tr(Cov\left(r\right)+Cov\left(g\right)-2{\left(Cov\left(r\right)Cov\left(g\right)\right)}^{1/2})$$

where μ(*r*) and μ(*g*) refer to the mean score of the training images and the generated images. *Cov* represents the covariance matrices, and *Tr* stands for the trace linear algebra operation. Since the scores (*r* and *g*) are a vector of observations, the *Cov* and *Tr* operation both return a scalar value. We randomly selected 798 images from the training datasets and 798 from the generated datasets and then substitute their evaluation score to (6). The FD value turned out to be 0.05634. As a baseline for comparison, the training set was randomly partitioned into two splits each with 798 phantoms. Equation (6) yielded an FD of 0.00662 for the two training sets distributions. Therefore, the difference between the two datasets is less than an order of magnitude which can be usually considered a fairly good result, to the best of our knowledge.

**Figure 6:**
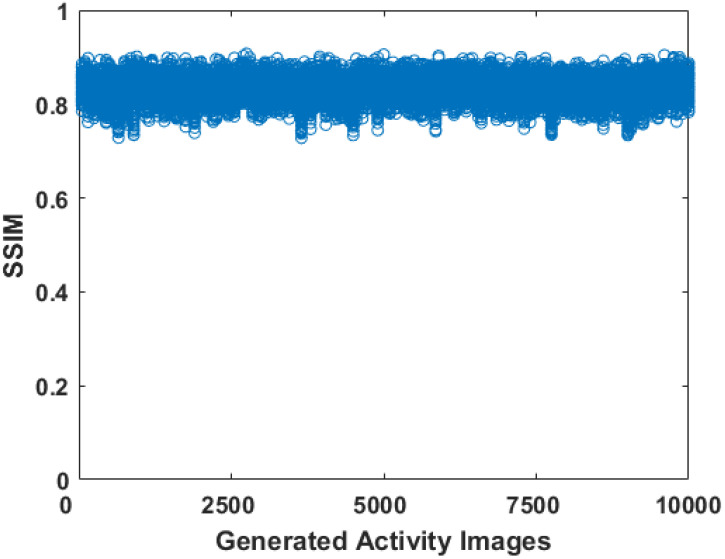
The SSIM values. The transverse axis represents the 10,000 generated phantoms. The vertical axis represents the SSIM values when comparing training phantoms with each generated phantom.

**Figure 7:**
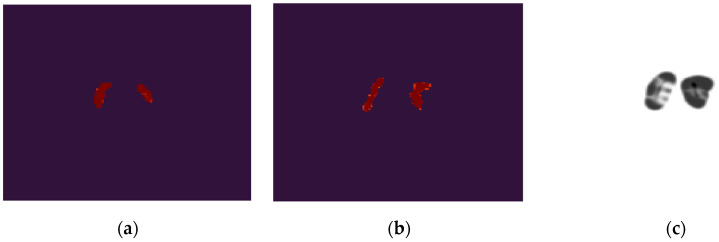
Study of the case of the smallest SSIM. (**a**) Shows the striatal region in a training phantom; (**b**) Shows the striatal region in a generated phantom; (**c**) Shows the local SSIM map when comparing (**a**) and (**b**). Note (**a**,**b**) were from different patients and could be in a different slice position.

Finally, the structural similarity (SSIM) index was employed to measure the similarity of the striatal region between the generated phantoms and the training phantoms. The SSIM is a metric that assesses the impact of three characteristics including luminance, contrast, and structure. The value of SSIM is in the range from 0 to 1, wherein 1 represents two completely identical objects. An ideal result will be that the SSIM in all generated phantoms remains a high value (e.g., >0.75), but unequal to one, meaning that the striata in the generated phantoms are in high similarity to, but different from, any in the training dataset. We compared each striatal region from the generated dataset with each in the training dataset. The SSIM values for a total of 10,000 × 1597 comparisons are presented in Figure 6, with the smallest value 0.7317 and the largest value 0.9074. We also illustrated the striatal images from the generated dataset and the training dataset for which the smallest SSIM was obtained, as well as the map showing the local values of SSIM in Figure 7. Small values of local SSIM appear as dark pixels in the local SSIM map, and a region with small local SSIM corresponds to an area having a relatively large difference between the two images. Since the work of this paper is limited to 2D, it is estimated that these small SSIM values derived mainly from significantly different trans-axial slices.

**Figure 8:**
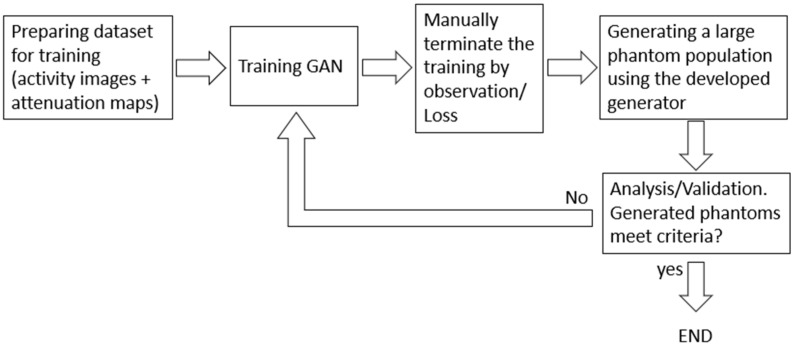
Flow chart—training a GAN for generating brain phantoms for SPECT research.

In Figure 8, we presented a flow chart to illustrate the entire procedures as to how we performed the training of GAN in this paper. Thus, readers will be easily able to follow these steps to develop GANs using their own training data.

## 4. Discussion

The goal of present work is to generate virtual datasets that are similar to those in the training dataset in the meantime, with more variations. The current generative network can produce 2D brain phantoms containing a pair of activity images and a corresponding attenuation map that mimic the uptake of [^123^I] ioflupane in brains of PD patients. The image matrix is 128 by 128. Such resolution is adequate for SPECT image research since the SPECT spatial resolution is usually 10–20 mm only (PET spatial resolution may reach 3–5 mm). The generated phantom can be used to simulate SPECT examination to evaluate other conventional or AI-based methods for PD research. The simulated projection data and the phantoms can be used together to train ML-based image reconstruction algorithms, or to evaluate quantitative accuracy of OSEM reconstruction with different compensation techniques [37]. Finally, the reconstructed images can then be used to develop ML methods for diagnosis or predicting outcomes of PD [38]. The activity level in the generated phantom can also be easily scaled to mimic the uptake of other tracers that target striatum region, such as ^11^C-RTI-32 [39] and [^99m^Tc]TRODAT [40], or be adjusted using image processing techniques to reflect PD stages. Meanwhile, the generated attenuation map can also be scaled to match the corresponding photon energy. In the current work, the generated skull in the attenuation map was considered solid and without marrow, but is adequate for modelling the attenuation because there is no tracer uptake in the skull.

It is difficult to evaluate the accuracy and variation of the produced phantoms since there is are no existing methods to validate a population of generated phantoms because they do not belong to any human being. Since there is no ground truth, conventional figure-of-merit such as mean-square error cannot be used. The results must be evaluated using statistical analysis by comparing the training data with the generated data. We presented several statistical analyses, including histogram of evaluation score, Fréchet distance, and SSIM in the Results. Alternatively, one can employ human observers and ROC analysis to investigate if humans can separate the two datasets. However, this will be subject to the number of phantoms being reviewed in limited time and the bias of the reviewers.

Regarding the next step, we will run Monte Carlo simulations to acquire SPECT data of the generated phantoms. The paired SPECT data and phantoms will then be used to develop deep learning models for image reconstruction, where phantoms will be serving the desired output of the neural network. On the other hand, the generated phantoms are 2D slices that can be used for 2D applications only, which is one of the limitations of the present work. However, the success encourages us to extend this work to 3D. Therefore, the 3D GAN technique will be explored which is expected to involve 3D convolution and transposed convolution computation, and the number of voxels in 3D phantoms will be tremendously increased. This will be a big challenge for us and for all, since the GAN has been known to be unstable when producing large images. More advanced network architecture and training techniques, such as the progressively growing GAN (PGGAN) ([29]), will be tested (PGGAN has been demonstrated to successfully produce 1280 by 1024 2D images). Finally, we will also try introducing more patient information into the GAN development such as gender and health conditions, so the generator will be able to produce specific phantom classes.

## 5. Conclusions

The study in this paper demonstrates that GAN can be used to generate digital phantoms that mimic the imaging of dopamine transporter and the corresponding attenuation maps. The generated phantoms can be used in Monte Carlo simulation to generate realistic projection data, so ML-based SPECT image reconstruction [41] and other applications [42] can be developed with known truth. Each generated phantom contains an activity image and a corresponding attenuation map. A few analytical methods have been used to evaluate the generated phantoms. Statistical results show that the similarity between the generated phantoms and the training dataset is high. With the developed generative network, one can produce an unlimited number of digital phantoms serving as the training data, to develop other ML-based SPECT imaging applications. Thus, the overfitting issue that widely exists in AI medical imaging can be effectively relieved.

## 6. Patents

A patent on the GAN-based methodology resulting from the work reported in this manuscript has been filed.

## Data Availability

Not applicable.
